# The progress and prospect of natural components in rhubarb (*Rheum ribes L.*) in the treatment of renal fibrosis

**DOI:** 10.3389/fphar.2022.919967

**Published:** 2022-08-29

**Authors:** Yangyang Wang, Fangwei Yu, Ao Li, Zijia He, Caiyan Qu, Caiying He, Xiao Ma, Huakui Zhan

**Affiliations:** ^1^ Clinical School of Medicine, Chengdu University of Traditional Chinese Medicine, Chengdu, China; ^2^ State Key Laboratory of Southwestern Chinese Medicine Resources, School of Pharmacy, Chengdu University of Traditional Chinese Medicine, Chengdu, China; ^3^ Affiliated Hospital of Chengdu University of Traditional Chinese Medicine-Sichuan Provincial Hospital of Traditional Chinese Medicine, Chengdu, China

**Keywords:** renal fibrosis, R. ribes L., inflammation, ECM, TGF-β, emodin

## Abstract

**Background:** Renal fibrosis is a key pathological change that occurs in the progression of almost all chronic kidney diseases . CKD has the characteristics of high morbidity and mortality. Its prevalence is increasing each year on a global scale, which seriously affects people’s health and quality of life. Natural products have been used for new drug development and disease treatment for many years. The abundant natural products in *R. ribes L*. can intervene in the process of renal fibrosis in different ways and have considerable therapeutic prospects.

**Purpose:** The etiology and pathology of renal fibrosis were analyzed, and the different ways in which the natural components of *R. ribes L*. can intervene and provide curative effects on the process of renal fibrosis were summarized. Methods: Electronic databases, such as PubMed, Life Science, MEDLINE, and Web of Science, were searched using the keywords ‘*R. ribes L*.’, ‘kidney fibrosis’, ‘emodin’ and ‘rhein’, and the various ways in which the natural ingredients protect against renal fibrosis were collected and sorted out.

**Results:** We analyzed several factors that play a leading role in the pathogenesis of renal fibrosis, such as the mechanism of the TGF-β/Smad and Wnt/β-catenin signaling pathways. Additionally, we reviewed the progress of the treatment of renal fibrosis with natural components in *R. ribes L*. and the intervention mechanism of the crucial therapeutic targets.

**Conclusion:** The natural components of *R. ribes L*. have a wide range of intervention effects on renal fibrosis targets, which provides new ideas for the development of new anti-kidney fibrosis drugs.

## 1 Introduction

Renal fibrosis is a common pathological change in the end stage of all chronic kidney diseases (CKDs) (Loboda et al., 2016). CKD has high morbidity and mortality (Black et al., 2018). Primary glomerular disease, diabetes and hypertension are major contributors to CKD (Webster et al., 2017). With the increasing incidence of diabetes and hypertension and the aging of the population, the prevalence of CKD has increased (Morgado-Pascual et al., 2018). Excessive deposition of the extracellular matrix (ECM) is the basic feature of renal fibrosis (Ma and Meng, 2019). Inflammation, key cytokines (such as TGF-β, Wnt, etc.), the extracellular matrix, autophagy, apoptosis, and epigenetic modifications have all been found to be associated with renal fibrosis (Lv et al., 2018; Morgado-Pascual et al., 2018; Zhao et al., 2019). At present, it is vital and imminent to discover new anti-kidney fibrosis drugs. Since ancient times, many valuable medicines have been discovered in nature (Cragg and Newman, 2013). *R. ribes L*. is a heat-clearing medicine in Chinese herbal medicine. The Chinese medicine *R. ribes L*. has achieved good effects in the treatment of many diseases. According to multiple reports, natural components, such as emodin, rhein, and aloe-emodin, are contained in *R. ribes L*. These components can improve renal fibrosis by regulating the above signaling pathways and the processes of oxidative stress and inflammation (Isaka, 2018; Lv et al., 2018). With the development of analysis and calculation techniques, new methods for processing natural products with complex structures have emerged (Thomford et al., 2018). This provides technical assistance for drug development. We will review the intervention effects of the natural components in *R. ribes L*. on certain key signaling pathways, explain the mechanism of the natural components in *R. ribes L*. to treat renal fibrosis, and explore new ideas for the development of renal fibrosis drugs.

## 2 Overview of the process of renal fibrosis

According to recent studies, the main cellular and molecular mechanisms involved in renal fibrosis include continuous stimulation of inflammatory cells, myofibroblast formation, massive extracellular matrix deposition (ECM), renal tubular atrophy and sparse microvascular networks (Liu, 2011). It is well known that the ECM is the most critical step in the renal fibrosis process, and myofibroblasts are the main component of the ECM. A large number of studies have shown that renal innate cells (including renal interstitial fibroblasts, pericytes, epithelial cells, and endothelial cells) are the main source of myofibroblasts (Sun Y. B. et al., 2016). In addition, studies have confirmed that some bone marrow-derived cells (such as phagocytes and T cells) can also be transformed into myofibroblasts under certain conditions (Meng et al., 2014). The above process is called cell phenotypic transformation. During kidney injury, fibroblasts activate and proliferate, becoming the main source of myofibroblasts (Liu, 2011). The transformation process of epithelial cells is called epithelial-mesenchymal transition (EMT). Refers to the phenotypic characteristics of myofibroblasts acquired by epithelial cells. These are characterized by increased expression of α-SMA, vimentin, and 1 type collagen (Cruz-Solbes and Youker, 2017). Similarly, endothelial cells can acquire mesenchymal or myofibroblast phenotypic characteristics after deadherence, and this process is termed the endothelial-mesenchymal transition (EndMT). The transformed endothelial cells show increased expression of FSP-1, ɑ-SMA, fibronectin, vimentin, and collagen types I and III (He et al., 2013). Pericytes are a type of mesenchymal cell that are partially or fully embedded in the capillary basement membrane. These cells play a role in maintaining capillary homeostasis and regulating blood flow. After renal injury, pericytes are separated from capillary endothelial cells through pericyte-myofibroblast transformation. This results in sparse capillaries and affects renal function (Kramann and Humphreys, 2014). The above process leads to renal interstitial fibrosis, podocyte lesions or glomerulosclerosis.

Inflammation is a key driver of renal fibrosis and plays a leading role in the transformation of cellular phenotypes (Meng et al., 2014). Long-term stimuli from damage to the body (such as proteinuria, hypertension, hyperglycemia, hypoxia, etc.) can lead to renal tissue damage, which in turn induces the inflammatory response of renal innate cells (Morgado-Pascual et al., 2018). Inflammatory cells, such as macrophages, lymphocytes, dendritic cells, and mast cells, aggregate to secrete cytokines and chemokines. These cytokines and chemokines aggregate inflammatory cells and induce the renal innate cell phenotype transformation. Damaged epithelial cells secrete cytokines, such as TGF-β and Wnt, and become proinflammatory cells, aggravating the inflammatory response. Activated endothelial cells and pericytes undergo phenotypic transformation. This causes capillary damage and the subsequent hypoxia, which further aggravate renal injury (Yuan et al., 2019). In addition, cytokines secreted by inflammatory cells, damaged epithelial cells, and myofibroblasts are also key factors in accelerating renal fibrosis. For example, TGF-β can promote renal fibrosis by activating the Smad or non-Smad signaling pathway. Wnt aggravates renal fibrosis by activating the Wnt/β-catenin signaling pathway. PDGF can promote the production of inflammatory mediators and accelerate the accumulation of ECM(Tan et al., 2014; Meng, 2019).

## 3 Clinical study of natural components in *R. ribes L*

As a traditional Chinese medicine, *R. ribes L*. is often used as a laxative to treat constipation (Gao et al., 2021). Modern clinical studies have demonstrate that *R. ribes L*. has considerable curative effects on some common clinical diseases. For example, the clinical studies of Wang et al. have indicated that *R. ribes L*. combined with early enteral nutrition can significantly improve gastrointestinal function, inhibit systemic inflammation, and reduce liver and kidney damage in patients with severe pancreatitis (Wan et al., 2014). In a clinical randomized controlled study by Liu et al., it was indicated that *R. ribes L*. can reduce blood lipid levels in patients with atherosclerosis and improve vascular endothelial function (Liu et al., 2007). To date, few clinical studies have confirmed that *R. ribes L*. has a therapeutic effect on renal fibrosis, but some clinical studies have confirmed that *R. ribes L*. has a certain intervention effect on renal disease. For example, clinical studies conducted by Irfan A Khan et al. confirmed that *R. ribes L*. has a significant effect on the conservative treatment of patients with advanced chronic kidney disease (Khan et al., 2014). The study by Li et al. confirmed that *R. ribes L*. can effectively prevent disease progression in patients with chronic renal failure (Li and Liu, 1991). Therefore, the author believes that the clinical treatment of *R. ribes L*. for renal fibrosis may also have certain potential.

The most abundant chemical constituents in *R. ribes L*. are anthraquinone compounds, mainly rhein, chrysophanol, emodin and aloe-emodin (Aichner and Ganzera, 2015). Modern pharmacological studies have confirmed that the rich natural extracts in *R. ribes L*., such as emodin and rhein, have anti-tissue fibrosis effects (Sun H. et al., 2016; Zhao et al., 2019). This article will mainly discuss the feasibility of *R. ribes L*. as a clinical candidate drug in the treatment of renal fibrosis by describing the antagonistic effect of the anthraquinones in *R. ribes L*. on renal fibrosis.

## 4 Pathogenesis of renal fibrosis

### 4.1 Inflammation

**FIGURE 1:**
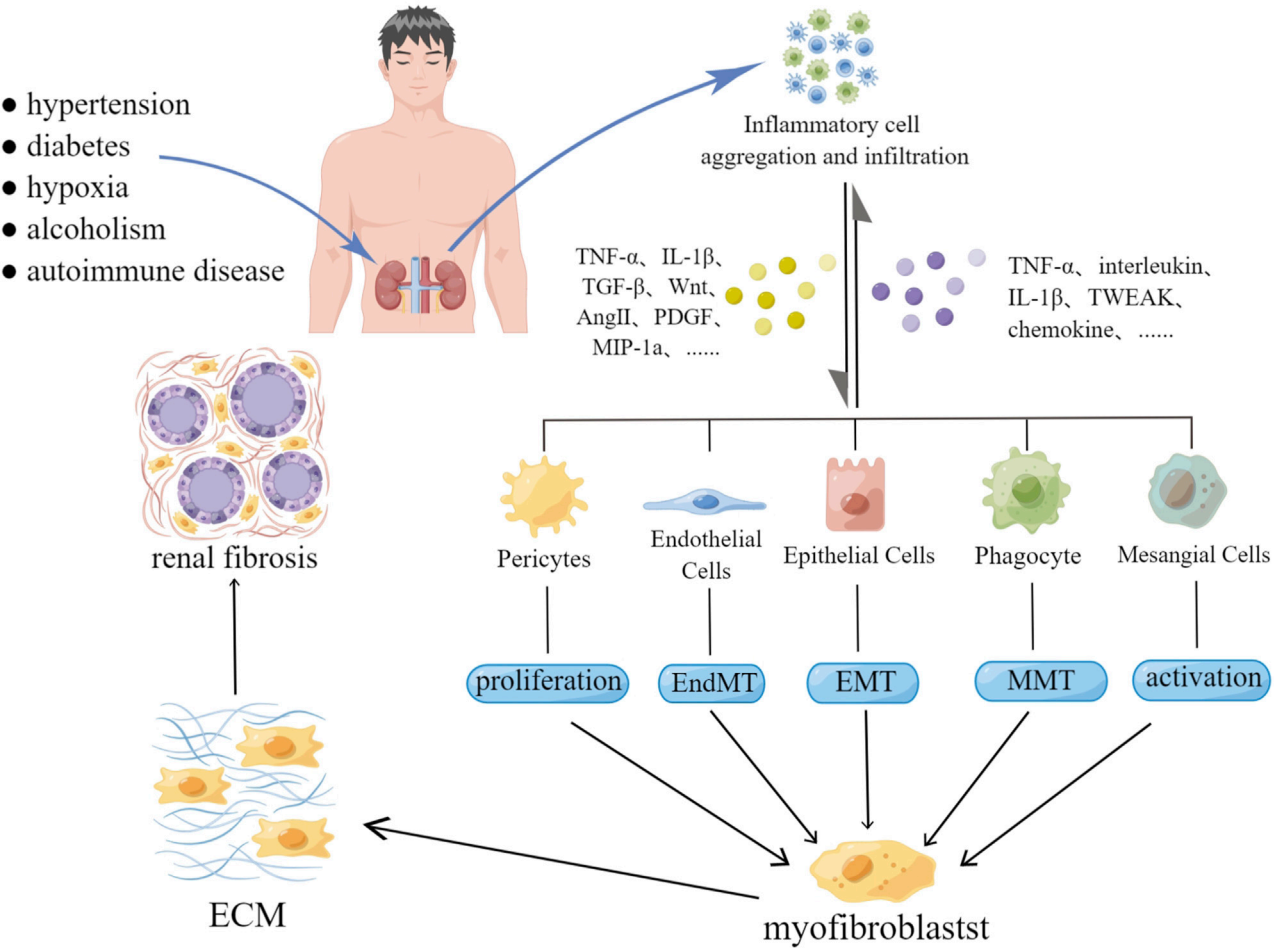
Inflammation and renal fibrosis.

Inflammation is the basis of renal fibrosis. Autoimmune diseases, high blood pressure, diabetes, obesity, drug damage, and poor lifestyle habits (such as alcoholism) are all common causes of kidney damage (Schaeffner and Ritz, 2012; Kurts et al., 2013; Mennuni et al., 2014; Tonneijck et al., 2017; Lakkis and Weir, 2018). Stimulated by the damage caused by immune and nonimmune factors, the inflammatory response of the kidneys acts to promote regeneration and repair (Tecklenborg et al., 2018). However, at the same time, damaged tissues release a large number of cytokines and inflammatory mediators, which continuously activate inflammatory cells and renal innate cells. Additionally, they promote cell phenotypic transformation, myofibroblast accumulation, and ECM processes, resulting in kidney fibrosis (Black et al., 2019). The mechanism of action of inflammation is shown in (Figure 1).

In renal injury, both inflammatory cells and renal innate cells are the driving forces that exacerbate inflammation and fibrosis. The first driving force is the profibrotic and proinflammatory effects of inflammatory cells. After early kidney injury, under the action of chemokines and cytokines, inflammatory cells (such as T cells, macrophages, fibroblasts, and mast cells) are recruited to the kidney and release profibrotic factors and cytokines. This further aggravates the inflammatory response (Meng, 2019). Stimulated inflammatory cells secrete TNF-α, IL-1β, TGF-β, Wnt, Ang II, PDGF, collagen, MIP-1a and other factors. These factors are involved in renal fibrosis and aggravate the inflammatory response (Chesney and Bucala, 2000; Duffield, 2010; Tang et al., 2019). Under the action of chemokines, growth factors and a series of enzymes, mast cells release inflammatory factors and profibrotic factors and activate the Ang II signaling pathway to play a profibrotic role (Meng et al., 2014).

The second driving force is the role of renal innate cells. During renal injury, tubular epithelial cells transform into a secretory phenotype. They secrete and activate proinflammatory cytokines, such as TNF-α, interleukin, IL-1β, and TWEAK, and aggravate inflammatory cell infiltration. In addition, epithelial cells secrete chemokines and adhesion molecules to promote the aggregation of inflammatory cells, which is also one of the important causes of kidney inflammation aggravation (Liu et al., 2018). Continued stimulation induces the EMT with the aim of repairing damage through collagen production by myofibroblasts. Moreover, under the stimulation of factors such as Ang II, epithelial cells secrete TGF-β. Due to persistent inflammatory stimulation and the long-term effects of cytokines such as TGF-β, kidney fibrosis occurs (Macconi et al., 2014; Cruz-Solbes and Youker, 2017). The second contributor is the role of mesangial cells. Injured mesangial cells produce a variety of chemokines, cytokines, and proinflammatory mediators, which activate inflammation and damage the endothelial cell barrier (Schlöndorff and Banas, 2009). Finally, endothelial cells play a role. After kidney injury, endothelial cells activate inflammatory responses and recruit inflammatory cells. The accumulation of inflammatory cells increases hemodynamic resistance, causes local hypoxia and induces endothelial cell apoptosis. These effects eventually lead to increased capillary permeability and further aggravate hypoxia (Kelly et al., 2009; Guerrot et al., 2012).

Overall, renal injury is the beginning of renal fibrosis, and persistent inflammatory stimulation is the basis and driving force for the progression and aggravation of renal fibrosis.

After kidney injury, the kidney triggers an inflammatory response to promote repair and regeneration. Inflammatory cells secrete chemokines, inflammatory factors and cytokines, aggravating the inflammatory response. Simultaneously activate renal innate cells to secrete cytokines and pro-inflammatory mediators. Persistent inflammation and cytokines stimulate the proliferation and differentiation of pericytes and mesangial cells, and induce EMT, EndMT, and MMT, resulting in an increase and accumulation of fibroblasts. Initiates ECM and promotes renal fibrosis.

### 4.2 Formation of the fibrous matrix

#### 4.2.1 The role of TGF-β

TGF-β is a growth factor that is secreted by most cells of the body. There are many different isoforms in the large TGF-β protein family. All of these members play an important role in regulating the growth, development and differentiation of cells in the body and are closely related to ligand secretion, ECM and presignaling activation (Massagué, 1998; Tzavlaki and Moustakas, 2020).

The TGF-β/Smad signaling pathway is closely related to diseases such as tissue fibrosis and cancer and has been a hot research topic in recent years (Blobe et al., 2000). TGF-β1 is the isoform that is most closely associated with tissue fibrosis (Tang R. et al., 2020). During renal fibrosis, TGF-β1 mainly activates downstream Smad3 signaling. After the binding of Smad3 and Smad4, they enter the nucleus and directly mediate renal fibrosis by promoting DNA methylation, reducing histone acetylation, and inducing miRNA transcription (Zhong et al., 2011; Tampe et al., 2014; Smith et al., 2019). In addition to the canonical TGF-β/Smad signaling pathway, TGF-β1 can also jointly mediate renal fibrosis by activating other signaling pathways, such as the MAPK and PI3K/Akt/mTOR pathways (Zhang, 2017; Nickel et al., 2018).

TGF-β also plays an important role in the phenotypic transformation of cells. Endothelial cells, epithelial cells, and macrophages can also transform into myofibroblasts under continuous stimulation of TGF-β1. This is a process that aggravates fibrous deposition and promotes renal fibrosis. Most studies have shown that TGF-β1 is mainly associated with EMT, and EndMT is mainly induced by TGF-β2 (Duffield, 2010; He et al., 2013; Cruz-Solbes and Youker, 2017). In addition, the secretion of TGF-β by mesangial cells under inflammatory stimulation induces mesangial cell proliferation and ECM, which further aggravates renal fibrosis (Zhao, 2019). In recent years, Wu et al. also confirmed that TGF-β1 can promote the differentiation of pericytes into myofibroblasts (Wu et al., 2013). TGF-β is also a key factor in activating myofibroblasts and inducing their proliferation and differentiation. The accumulation of fibroblasts causes ECM accumulation and renal fibrosis (Yuan et al., 2019).

#### 4.2.2 The role of wnt

**FIGURE 2:**
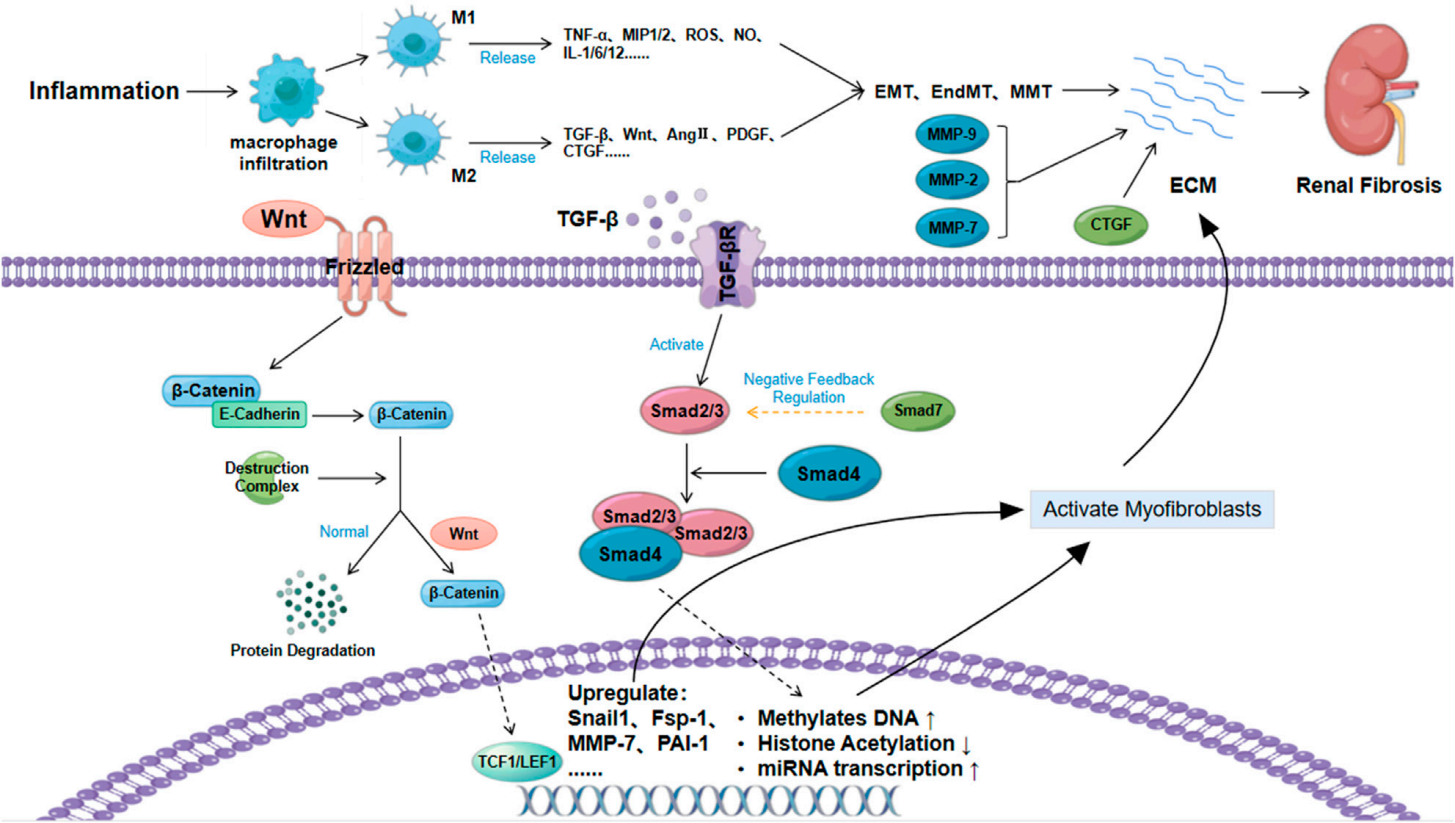
Formation of the fibrous matrix.

The secreted growth factor protein Wnt is closely related to cell renewal, proliferation and differentiation (Majidinia et al., 2018). The typical Wnt signaling pathway is the Wnt/β-catenin signaling pathway, which is relatively conserved (Steinhart and Angers, 2018). As a key nuclear effector of the Wnt signaling pathway, β-catenin exhibits different biological effects in the cell membrane, cytoplasm and nucleus (Valenta et al., 2012). β-Catenin forms a molecular complex with cadherin (usually E-cadherin), and the deadhesion of the E-cadherin/β-catenin complex plays an important role in the EMT (Tian et al., 2011). Normally, after E-cadherin/β-catenin deadhesion, free β-catenin is degraded (phosphorylated) by the destruction complex (Guo et al., 2012). Abnormal Wnt protein expression can increase the amount of free β-catenin by acting on the Twist and Slug genes to inhibit the transcription of E-cadherin. Then, it can block the degradation of β-catenin by acting on β-catenin to destroy the complex (Heuberger and Birchmeier, 2010; Valenta et al., 2012). The excessive accumulation of free β-catenin causes free β-catenin to translocate to the nucleus to activate TCF and LEF. This then induces the expression of fibronectin, Fsp-1, Snail genes, MMP-7, and PAI-1, promotes myofibroblast activation, accelerates ECM accumulation and promotes kidney fibrosis (Stemmer et al., 2008; Hao et al., 2011; Tan and Liu, 2012; Zuo and Liu, 2018). In addition to the Wnt proteins, TGF-β1, Akt and other factors can also induce renal fibrosis by activating β-catenin, as shown in Figure 2 (Fang et al., 2007; Wang et al., 2011).

#### 4.2.3 The role of the extracellular matrix

The extracellular matrix not only acts as a scaffold to maintain cell stability but also plays a key role in inducing renal fibrosis. ECM is the main pathological factor of fibrosis. MMP plays an important role in the process of ECM accumulation. MMP is a metalloproteinase that can degrade collagen, and TIMP has an inhibitory effect on its activity (Bülow and Boor, 2019). Current studies have found that several subtypes of the MMP family, such as MMP-2, MMP-7, and MMP-9, are closely related to fibrosis (Cheng and Lovett, 2003; Wang et al., 2010; Ke et al., 2017). MMP-2 causes renal tubular damage and fibrosis by disrupting the integrity of the basement membrane. In addition, the synergistic effect of MMP-2 and TGF-β1 to promote the EMT has also been confirmed (Cheng and Lovett, 2003; Wozniak et al., 2021). MMP-7 can degrade E-cadherin, release β-catenin, reduce cell adhesion and accelerate the EMT (Wozniak et al., 2021). MMP-9 promotes the recruitment of macrophages and induces the EMT by cleaving osteopontin. In addition, MMP-9 also promotes the EndMT, which is mainly accomplished through the Notch signaling pathway (Zhao et al., 2017; Wozniak et al., 2021).

In addition to MMP, the role of CTGF in the cell matrix in fibrosis cannot be ignored, and this process is mainly accomplished by regulating other growth factor-mediated fibrosis-related signaling pathways (Toda et al., 2018). A large number of studies have confirmed that the expression of CTGF in patients with renal fibrosis is significantly increased. CTGF can not only promote EMT, recruit inflammatory cells, and induce ECM but also act as a downstream molecule of the TGF-β/Smad signaling pathway to increase renal fibrosis through multiple pathways (Yin and Liu, 2019). Fibrosis therapy treatments targeting CTGF have been shown to have certain potential (Phanish et al., 2010).

ECM deposition is the most critical pathological change in renal fibrosis. TGF-β, Wnt and extracellular matrix are the main factors affecting the ECM. TGF-β can directly synthesize fibrous matrix by inducing epigenetic modification and miRNA expression through the TGF-β/Smad signaling pathway, or it can induce the accumulation of myofibroblasts by inducing cell phenotypic transformation, and indirectly lead to ECM. Wnt can activate myofibroblast activation by blocking β-catenin degradation. The abnormal expression of metalloproteinases and CTGF in the extracellular matrix under the condition of renal injury is also an important reason for aggravating renal fibrosis.

### 4.3 Apoptosis and autophagy signal transduction

#### 4.3.1 Regulation of apoptosis

**FIGURE 3:**
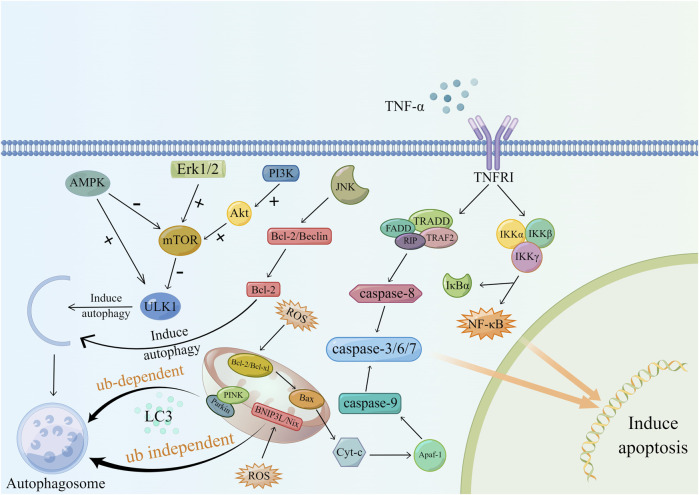
Apoptosis regulation and autophagy.

Apoptosis is a type of programmed cell death that plays a key regulatory role in the development of kidney disease. Apoptosis can be regulated in two ways, endogenous and exogenous, both of which can cause different degrees of renal damage when unregulated (Savill, 1994). Its mechanism is shown in (Figure 3).

Animal experiments have demonstrated the correlation between renal fibrosis and apoptosis and that mitochondrial damage mediated by oxidative stress is one of the important causes of uncontrolled apoptosis (Xu et al., 2013). In CKD patients, changes in renal hemodynamics and the continuous stimulation of inflammation lead to increased mitochondrial anaerobic metabolism, lipid deposition in the cytoplasm, and changes in mitochondrial membrane permeability (Martínez-Klimova et al., 2020). The increased release of ROS from damaged mitochondria further increases mitochondrial membrane permeability. Cytochrome-C (cytochrome C), Smac/DIABLO and other proapoptotic factors are released, and then these factors activate caspase-9, caspase-3 and caspase-7 through anti-perinuclear factor-1 (Apaf-1) to initiate apoptosis (Yang et al., 2006). Studies have shown that excessive apoptosis in the renal parenchyma in the short term can cause glomerular and tubular atrophy, while long-term tubular atrophy provides a suitable microenvironment for the formation of fibrosis (Ortiz et al., 1996).

Second, another related factor that regulates apoptosis is TNF-α. Macrophages and renal tubular epithelial cells are major sources of TNF-α. Studies by R. Misseri et al. showed that elevated levels of TNF-α protein in obstructed kidneys can induce apoptosis in renal tubular cells (Misseri and Meldrum, 2005; Misseri et al., 2005). TNF-α-mediated apoptosis includes two regulatory pathways. After TNF-α binds to cell membrane surface receptors, it forms a complex with TRADD and then couples with FADD to activate caspase-8. In addition, TNF-α can also activate NF-κB through the IKK complex. However, NF-κB has a bidirectional regulatory effect on apoptosis, and its proapoptotic or antiapoptotic function depends on the surrounding environment of the cell (Chinnaiyan et al., 1995; Hsu et al., 1995; Misseri and Meldrum, 2005; Hayden and Ghosh, 2014).

#### 4.3.2 Autophagy

Autophagy is a lysosomal degradation process that can occur in different types of kidney cells and plays an important role in maintaining cell stability (Zhao et al., 2019). Studies have shown that the regulation of autophagy in the process of renal fibrosis is bidirectional. Autophagy can mediate collagen degradation and inhibit renal fibrosis. Moreover, persistent activation of autophagy can lead to tubular atrophy and promote fibrosis (Kim et al., 2012; Livingston et al., 2016). Mitophagy is the molecular basis of cell-selective autophagy, and it includes ubiquitin (Ub)-dependent and Ub-independent pathways (Tang C. et al., 2020). During mitochondrial damage, PINK binds to Parkin and aggregates autophagy-related proteins, such as NBR1, through the polyubiquitination of Parkin. PINK then initiates autophagy by interacting with LC3. In the ubiquitin-independent pathway, mitochondrial hypoxia increases the expression of BNIP3L/Nix, which can also induce autophagy after binding to LC3 (Ding and Yin, 2012).

mTOR is a key regulator of autophagy, and its activation is related to signaling pathways mediated by factors such as MAPK, PI3K, and AMPK. In this process, the perception of the cell to changes in the nutritional and energy status plays a part in the selection role (Tang C. et al., 2020). AMPK, mTORC1, and ULK1 are known as the kinase triplet. When nutrients are abundant, mTOR is activated and inhibits ULK1 through phosphorylation. Then, it blocks the binding of AMPK to ULK1, thereby inhibiting autophagy. Upon starvation, ULK1 inhibition is released, and AMPK can activate autophagy by activating ULK1 (Kim and Guan, 2011; Dunlop and Tee, 2013). The signaling pathways related to MAPK and autophagy regulation include the MAPK/JNK signaling pathway and MAPK/Erk signaling pathway. JNK mainly relieves Beclin dependence and induces autophagy by destroying Bcl-2/Beclin and phosphorylating Bcl-2. Erk inhibits autophagy by activating mTOR. JNK and Erk have opposite regulatory effects on autophagy (Kim and Guan, 2015; Zhou Y. Y. et al., 2015). In addition, growth factors, such as insulin, can activate the PI3K/Akt/mTOR signaling pathway. Activated Akt can promote the dissociation of TSC1/2 and activate mTOR by targeting Rheb. Additionally, Akt can directly interact with mTOR to inhibit autophagy (Kim and Guan, 2015). The molecular mechanism of autophagy is shown in (Figure 3).

Hypoxia-induced mitochondrial damage underlies apoptosis and autophagy. After mitochondrial injury, pro-apoptotic factors and autophagy-related factors are expressed to initiate apoptosis and autophagy. In addition, activation of mTOR through MAPK/Erk, PI3K, AMPK and other related signaling pathways can promote autophagy. It has to be said that in the process of autophagy, JNK plays an inhibitory role.

### 4.4 Epigenetic modifications

#### 4.4.1 Deoxyribonucleic acid methylation

DNA methylation generally occurs on CpG islands located in the DNA promoter region. In this process, a methyl group is covalently added to the C5 position of the cytosine residue to achieve gene transcription silencing through physical obstruction (Larkin et al., 2018). Under normal circumstances, DNA methylation is in a dynamic state, maintaining stable intracellular gene transcription. Abnormal DNA methylation patterns can occur in most kidney diseases, such as AKI, DN, CKD and DKD. Regardless of the type of kidney damage, the end result is the induction of end-stage renal disease and renal fibrosis (Ding et al., 2021). The process of DNA methylation is regulated by DNA methyltransferase 1 (DNMT1). The main mechanism of this process is to induce chromatin conformational changes, thereby hindering the entry of proteins required for transcription and inhibiting transcription (Wanner and Bechtel-Walz, 2017). In the analysis of the whole genome and specific genes of CKD patients, it was found that the DNA methylation sites of CKD patients were different from those of nonrenal insufficiency patients. These differentiated genes (such as NOS3, TGFB3, and CARS2) are involved in the process of destroying renal function and inducing fibrosis. In addition, hypermethylation of these specific genes (such as RASAL1, Klotho, etc.) can also directly lead to severe renal fibrosis, suggesting that the steady-state imbalance of DNA methylation directly affects renal fibrosis progression (Chen et al., 2013; Wing et al., 2014; Mao, 2015).

#### 4.4.2 Histone modifications

Histones are highly conserved proteins in mammals and are the building blocks of nucleosomes. Histone modifications include acetylation, methylation, phosphorylation and other processes. Histone modifications include acetylation, methylation, phosphorylation and other processes. Renal fibrosis is mainly related to histone acetylation and methylation processes, which mainly occur on key amino acids in histone tails (Tampe and Zeisberg, 2014b; Ding et al., 2021).

Histone acetylation is generally associated with active gene transcription. It promotes gene transcription by reducing the net positive charge of histones and reducing the interaction between histones and DNA. This process is induced by histone acetyltransferases (HATs) and can be reversed by histone deacetylases (HADCs) (Morgado-Pascual et al., 2018). Most studies have found that the levels of HDAC1, HDAC2, and HDAC7 are increased in the kidneys of mice of the UUO model. This decreased histone acetylation and increased deacetylation. These effects resulted in gene transcriptional silencing and the induction of inflammation and renal fibrosis (Pang et al., 2009; Marumo et al., 2010). Various HDAC inhibitors (such as TSA, FK228, butyrate, MS-275, and valproic acid) can reverse the activation and proliferation of fibroblasts, tubular death and the EMT caused by abnormal HDAC expression. Fibrosis-related signaling pathways induced by factors such as TGF-β, EGRF, STAT3, and JNK/Notch2 can also be inhibited (Nie et al., 2020). This finding indicates that histone deacetylation is positively correlated with renal fibrosis and that reducing histone deacetylation can effectively alleviate renal fibrosis.

Another important genetic modification is histone methylation. Histone methylation can both activate and repress gene transcription, which is related to the position of the modified amino acid residues (Morgado-Pascual et al., 2018). In a study by Sun et al., TGF-β1 significantly increased the expression of ECM-related genes, such as collagen-1α1, CTGF, and PAI-1, by inducing histone lysine 4 methylation (H3K4me) and promoted fibrosis (Tampe and Zeisberg, 2014a).

## 5 Modern pharmacological studies of natural components in *R. ribes L*

**FIGURE 4:**
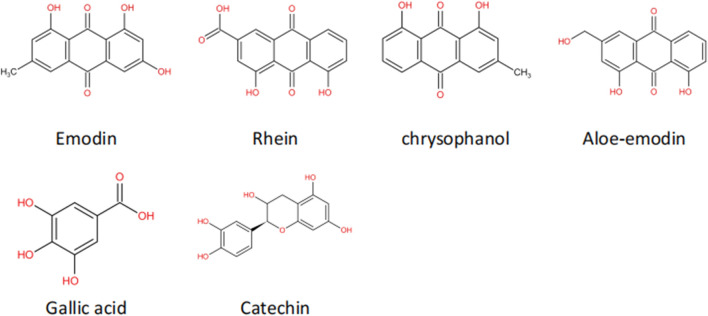
Molecular structure of natural ingredients.

Emodin is generally extracted from the rhizomes of plants. Studies have found that emodin has anticancer, anti-inflammatory, antibacterial, and antioxidant effects (Dong et al., 2016; Cui et al., 2020). Its molecular formula is shown in Figure 4. In recent years, studies have confirmed that in addition to the above effects, emodin also has an anti-tissue fibrosis effect (Zheng et al., 2021). Rhein is another anthraquinone compound with good lipophilicity (Zhou Y. X. et al., 2015). Modern pharmacological studies have found that rhein has anti-inflammatory, antitumor and antifibrotic effects and has certain protective effects in the liver and kidneys. The molecular schematic diagram of rhein is shown in Figure 4 (Sun H. et al., 2016; Li et al., 2021). Chrysophanol is derived from Polygonaceae plants. According to reports in recent years, chrysophanol has anti-inflammatory, antioxidant, anticancer, and neuroprotective effects (Xie et al., 2019; Yusuf et al., 2019). Chrysophanol also shows considerable curative effects in the fight against renal fibrosis, especially its inhibitory effect on the TGF-β/Smad signaling pathway. Aloe-emodin is not only rich in *R. ribes L*. but is also an effective ingredient in Chinese medicinal materials such as aloe and fleece-flower root. It has anticancer, antiviral, antibacterial, antiparasitic and other pharmacological effects (Dong et al., 2020).

## 6 Inhibitory effects of the natural ingredients on renal fibrosis

### 6.1Emodin

#### 6.1.1 Inhibition of the inflammatory response and podocyte injury

Zhu et al. reported that emodin at a concentration of 20 μM or 40 μM could inhibit lipopolysaccharide (LPS)-induced renal injury and increase the inflammatory response and interstitial fibrosis in mouse TECs. Moreover, emodin mediated the decrease in TNF-ɑ, IL-6 and TLR4 mRNA levels. This suggests that emodin can reduce inflammation and renal injury and inhibit renal fibrosis by inhibiting the LPS/TLR4/NF-κB signaling pathway (Zhu et al., 2012). A study by Chen et al. confirmed that emodin can inhibit high glucose-induced podocyte dysfunction and EMT. In addition, emodin decreased ILK and desmin expression in podocytes and increased renin expression. Therefore, emodin attenuates renal injury and restores podocyte function by inhibiting ILK expression (Chen et al., 2015).

#### 6.1.2 Inhibition of TGF-β

Ma et al. confirmed that 1 mg/kg emodin could improve renal function in 5/6 nephrectomy rats. In addition, rhubarb was also detected to reduce the levels of fibrosis-related molecules, such as TGF-β1 mRNA and FN, α-SMA, CTGF and Smurf2 proteins, while upregulating the level of Smad7. It has been shown that emodin can inhibit renal fibrosis by inhibiting the TGF-β/Smad signaling pathway (Ma et al., 2018). Yang et al. found that 20 μM emodin inhibited TGF-β1-induced activation of FN and ɑ-SMA in HK2 cells. When emodin was used in combination with HGF, the expression of TGF-β1 in UUO mice and in HK2 cells was decreased. This indicates that the combined use of HGF and emodin can inhibit renal fibrosis by inhibiting the TGF-β/Smad signaling pathway, and the effect is better than that of emodin alone (Yang et al., 2020). Yang et al. confirmed that emodin can inhibit the expression of TGF-β1 and FN by inhibiting the NF-κB signaling pathway (Yang et al., 2013). In addition, the study of Liu Wei et al. confirmed that emodin can improve TGF-β1-induced EMT. Downregulation of TGF-β, FN and α-SMA levels inhibits renal fibrosis (Liu W. et al., 2021).

#### 6.1.3 Regulation of autophagy and apoptosis

Liu Hong et al. confirmed that emodin could improve renal fibrosis, podocyte injury and epithelial cell apoptosis in streptozotocin (STZ)-treated rats. Experiments showed that emodin enhanced the protein expression of LC3-II/I, Beclin-1, and p-AMPK and inhibited the protein expression of p62 and p-mTOR. Therefore, emodin enhances podocyte autophagy, inhibits apoptosis, and alleviates renal fibrosis mainly by regulating the AMPK/mTOR signaling pathway (Liu H. et al., 2021). Liu Wei et al. confirmed that 100 μM emodin could inhibit and improve renal function in rats. Studies have shown that emodin induces the downregulation of p62 protein expression, while BMP-7, Beclin1, and LC3B protein expression is upregulated. It was demonstrated that emodin can clear injured renal cells by upregulating BMP-7-induced autophagy, alleviating EMT, maintaining renal homeostasis, and inhibiting fibrosis (Liu W. et al., 2021).

Chen et al. showed that emodin attenuates the hypoxia/reoxygenation (H/R)-induced loss of cell viability and apoptosis. Emodin induces an increase in Bcl-2 expression and a decrease in Bax expression. This reduces tubular apoptosis to protect the renal tubules from H/R damage (Chen et al., 2017). Tian et al. used KKay mice and found that 80 mg/kg/d emodin inhibited endoplasmic reticulum stress and podocyte apoptosis induced by HG. Furthermore, emodin treatment decreased the expression of endoplasmic reticulum kinase (P-PERK), P-eIF2α, ATF4 and CHOP. This suggests that emodin can antagonize the podocyte apoptosis that is induced by endoplasmic reticulum stress by inhibiting the PERK/eIF2α signaling pathway (Tian et al., 2018).

#### 6.1.4 Restoration of normal mesangial cell function

Yang et al. reported that emodin could alleviate high glucose-induced renal mesangial cell proliferation and glomerulosclerosis. Decreased levels of p65 and increased levels of IκB were detected in the study. Emodin can inhibit the proliferation of mesangial cells by inhibiting the NF-κB signaling pathway (Yang et al., 2013). Wang Rong et al. found that emodin can improve IL-1β-induced mesangial cell proliferation and extracellular matrix deposition. Experiments demonstrated that the upstream kinase P-MKK3/6 activity of p38 was inhibited. Emodin reduces renal fibrosis by inhibiting the p38/MAPK signaling pathway (Wang et al., 2007). Similarly, Li et al. also proposed that emodin can inhibit the abnormal proliferation of rat mesangial cells mediated by HG. The levels of the p38/MAPK downstream protein factors CREB and CTGF were decreased. It was confirmed that emodin can inhibit the proliferation of mesangial cells, which is mainly achieved by inhibiting the p38/MAPK signaling pathway (Li et al., 2009). The study by Xu et al. showed that emodin can also inhibit the abnormal proliferation of mesangial cells that is induced by HG by upregulating the levels of Bax and caspase. Emodin can also improve renal fibrosis by inducing the apoptosis of abnormally proliferating mesangial cells (Xu et al., 2016b). Gao et al. proposed that the abnormal proliferation and apoptotic inhibition of mesangial cells caused by HG may be related to the activation of CFLIP. After emodin treatment, the level of CFLIP decreased, apoptosis inhibition was relieved, and the expression of FN decreased. It was proven that the protective effect of emodin on the kidney tissue of DN mice may be related to the inhibition of CFLIP expression (Gao et al., 2014). Liu et al. found that emodin ameliorated high glucose-induced mesangial cell contractility deficiency and increased the glomerular filtration rate. It was confirmed that emodin activates PPARγ mRNA and reduces the expression of P38. This finding indicates that emodin improves the function of mesangial cells and glomeruli in diabetic nephropathy by inhibiting the P38/MAPK signaling pathway (Liu et al., 2009).

### 6.2 Rhein

#### 6.2.1 Inhibition of inflammation and oxidative stress

Rhein also has considerable efficacy in inhibiting kidney inflammation and oxidative stress. A study published in 2020 showed that rhein significantly inhibited the lipopolysaccharide-induced aggravation of kidney inflammation in 5/6 Nx rats and protected the function of damaged kidneys. Experiments detected that the expression of proinflammatory factors, such as TNF-ɑ, IL-6 and MCP-1, was inhibited. In particular, the level of phosphorylated IκBɑ was significantly reduced, and the mechanism by which rhein inhibits the inflammatory response and oxidative stress may act to block the activation of the NF-κB signaling pathway by inhibiting IκBɑ phosphorylation (Liu M. et al., 2021). Similarly, Chen et al. demonstrated that rhein attenuates the renal inflammatory response in CGN rats by inhibiting the activation of the NF-κB signaling pathway (Chen et al., 2022). Studies have reported that treatment with rhein in 5/6 Nx rats alleviated tubular damage, interstitial inflammation, and collagen deposition. By using SIRT3 knockout as a control experiment, it was confirmed that rhein inhibited Foxo3a expression and reduced ROS synthesis and oxidative stress. The pharmacological mechanism of rhein acts to inhibit the expression of the SIRT3/Foxo3a signaling pathway (Wu et al., 2020). The experiments of Chen Yakun et al. also found that UUO was accompanied by an increase in the phosphorylation level of STAT3, and the expression of ɑ-SMA and type I collagen was increased. The mRNA expression levels of STAT3 and ɑ-SMA were inhibited by rhein treatment. This also indicates that the inhibition of renal fibrosis by rhein may be related to the inhibition of STAT3 expression by rhein (Chen et al., 2019).

#### 6.2.2 Inhibition of TGF-β and wnt

In a study by Ho et al., rhein attenuated the collagen deposition caused by interstitial injury in UUO mice. Rhein inhibits the activation of the TGF-β/Smad signaling pathway by inhibiting the expression of TGF-β1 and TβRI and improves the deformation of renal tubular epithelial cells, tubular atrophy, ECM deposition and renal fibrosis in mice (He et al., 2011). Rhein also intervenes in the Wnt/β-catenin signaling pathway. In a study by Duan et al., rhein inhibited the expression of Wnt protein, β-catenin and GSK3b in the kidneys of db/db mice. Rhein can also increase the level of renin in podocytes and inhibit the ablation of podocyte foot processes, thereby maintaining podocyte stability (Duan et al., 2016).

#### 6.2.3 Regulation of epigenetic modifications

Klotho is a joint-regulatory gene that is involved in renal fibrosis. It can inhibit key signaling pathways that mediate fibrosis, such as TGF-β/Smad and Wnt/β-catenin. In a study by Zhang et al., rhein attenuated UUO treatment and TGF-β1-induced DNMT aberrations. Klotho inhibition regulates fibrosis-related signaling pathways by reversing Klotho methylation, improves abnormal pathological changes in renal tissue, and slows the process of renal fibrosis (Zhang et al., 2016). Klotho also mediates toll-like receptor 4 (TLR4) degradation, thereby reducing lipopolysaccharide (LPS)-induced acute inflammation. Studies have shown that the expression of the NF-κB signaling pathway and cytokines in mice treated with rhein was reduced, mainly because rhein relieved the inhibitory effect of LPS on Klotho (Bi et al., 2018).

#### 6.2.4 Apoptosis regulation

In the study of Chen et al., the expression of Bax was increased and the expression of Bcl2 was decreased in rats after UUO treatment. The ratio of Bax to Bcl2 was significantly decreased after treatment with emodin, indicating that emodin can regulate cell apoptosis (Chen et al., 2019). Xu et al. placed RMC cells in a high glucose environment and found that rhein can antagonize the abnormal proliferation of mesangial cells caused by HG. Rhein downregulated HG-induced collagen IV and laminin mRNA expression. Evidence suggests that this is achieved primarily by regulating apoptosis in abnormally proliferating mesangial cells (Xu et al., 2016a).

### 6.3 Chrysophanol

#### 6.3.1 Inhibition of TGF-β

A study by Dou et al. found that chrysophanol can downregulate the mRNA expression of TβRI, Smurf2, TGF-β1, FN, ɑ-SMA, collagen type I and collagen type III both *in vivo* and *in vitro*, thereby inhibiting renal fibrosis. Further studies confirmed that chrysophanol inhibits the activation of the TGF-β/Smad signaling pathway by inhibiting the expression of TβRI and Smurf2 and promoting the interaction between Smad7 and Smurf2 and TβRI (Dou et al., 2020). Guo Chuan et al. placed AB8/13 cells in a high-glucose environment. They found that HG induced the expression of Smad2 and Smad3 in podocytes and increased the levels of TNF-ɑ, IL-6, and IL-1β. After chrysophanol treatment, the TGF-β/Smad signaling pathway was inhibited, and the above inflammation- and fibrosis-related factors were inhibited (Guo et al., 2020).

#### 6.3.2 Inhibition of inflammation, apoptosis and oxidative stress

Another study demonstrated that chrysophanol alleviated cisplatin (CDDP)-induced acute kidney injury (AKI). Its pharmacological mechanism may primarily act through the inhibition of CDDP-induced oxidative stress, apoptosis and the NF-κB signaling pathway (Ma et al., 2021).

### 6.4 Aloe-emodin

#### 6.4.1 PI3K/Akt/mTOR

To verify the anti-renal fibrosis effect of aloe-emodin, Dou et al. constructed a model of renal fibrosis *in vitro* and *in vivo*. Studies have found that aloe-emodin can reduce the protein levels of PI3K, Akt and mTOR in UUO mice, improve biochemical indicators such as Scr and BUN in UUO mice, and inhibit the mRNA expression of TGF-β1, collagen type I, collagen type IV and FN. In the *in vitro* model, aloe-emodin blocked the activation of the PI3K/Akt/mTOR signaling pathway in TGF-β1-treated HK2 cells. The above findings all prove that aloe-emodin can inhibit the PI3K/Akt/mTOR signaling pathway and improve renal fibrosis (Dou et al., 2019).

### 6.5 Gallic acid

In addition to the above natural components, there are also some natural products with a lower content in rhubarb that also have a considerable intervention effect on renal fibrosis. For example, gallic acid can reduce the expression of fibrosis-related molecules, such as collagen and MMP-2, in the kidneys of rats induced by glyoxal. It can also reduce the level of serum markers such as Scr, protect renal function, and inhibit renal fibrosis (Yousuf and Vellaichamy, 2015). In addition, studies have reported that gallic acid can improve renal dysfunction and inflammatory responses in diabetic nephropathy rats. It can also reduce IL-1β, IL-6, TNF-α levels, and improve renal fibrosis by inhibiting p38 MAPK signaling pathway and NF-κB signaling pathway (Ahad et al., 2015).

### 6.6 Catechin

Muragundla et al. found that catechin can reduce ROS activity. This indicates that catechin can protect against renal function damage caused by CsA by inhibiting oxidative stress. Thus, it can inhibit renal interstitial fibrosis and reduce the degree of renal tubular atrophy (Anjaneyulu et al., 2003).

The natural components in rhubarb (such as rhein, emodin, chrysophanol, aloe-emodin, gallic acid and catechin) can be used for TGF-β/Smad, LPS/TLR4/NF-κB, Wnt/β, respectively. -Catenin, PI3K/Akt/mTOR and other signaling pathways achieve the purpose of inhibiting oxidative stress, regulating apoptosis and autophagy, and reducing inflammation. Ultimately, the anti-fibrotic effect is achieved.

## 7 Discussion and outlook

In recent years, there have been many studies on the effectiveness of natural components in *R. ribes L*. against clinical diseases. For instance, emodin has high potential for the treatment of atherosclerosis, cardiovascular disease, liver disease and cancer and has the advantages of multitarget, multiefficacy, and broad pharmacological effects (Hsu and Chung, 2012; Hu et al., 2020; Luo et al., 2021). Rhein has shown inhibitory effects against a variety of cancers, both in *in vitro* and *in vivo* models, and rhein can modulate different signaling cascades in cancer cells (Henamayee et al., 2020). Chrysophanol can effectively prevent and treat central nervous system diseases, such as stroke and cerebral hemorrhage (Li et al., 2019). Furthermore, many studies based on animal models have confirmed that the natural components in *R. ribes L*. can delay the process of renal fibrosis. Therefore, we reviewed the research progress of the natural components in the Chinese herbal medicine *R. ribes L*. in inhibiting renal fibrosis in recent years, considered its advantages and disadvantages, and summarized and discussed how to exert the therapeutic potential of this drug.

**FIGURE 5:**
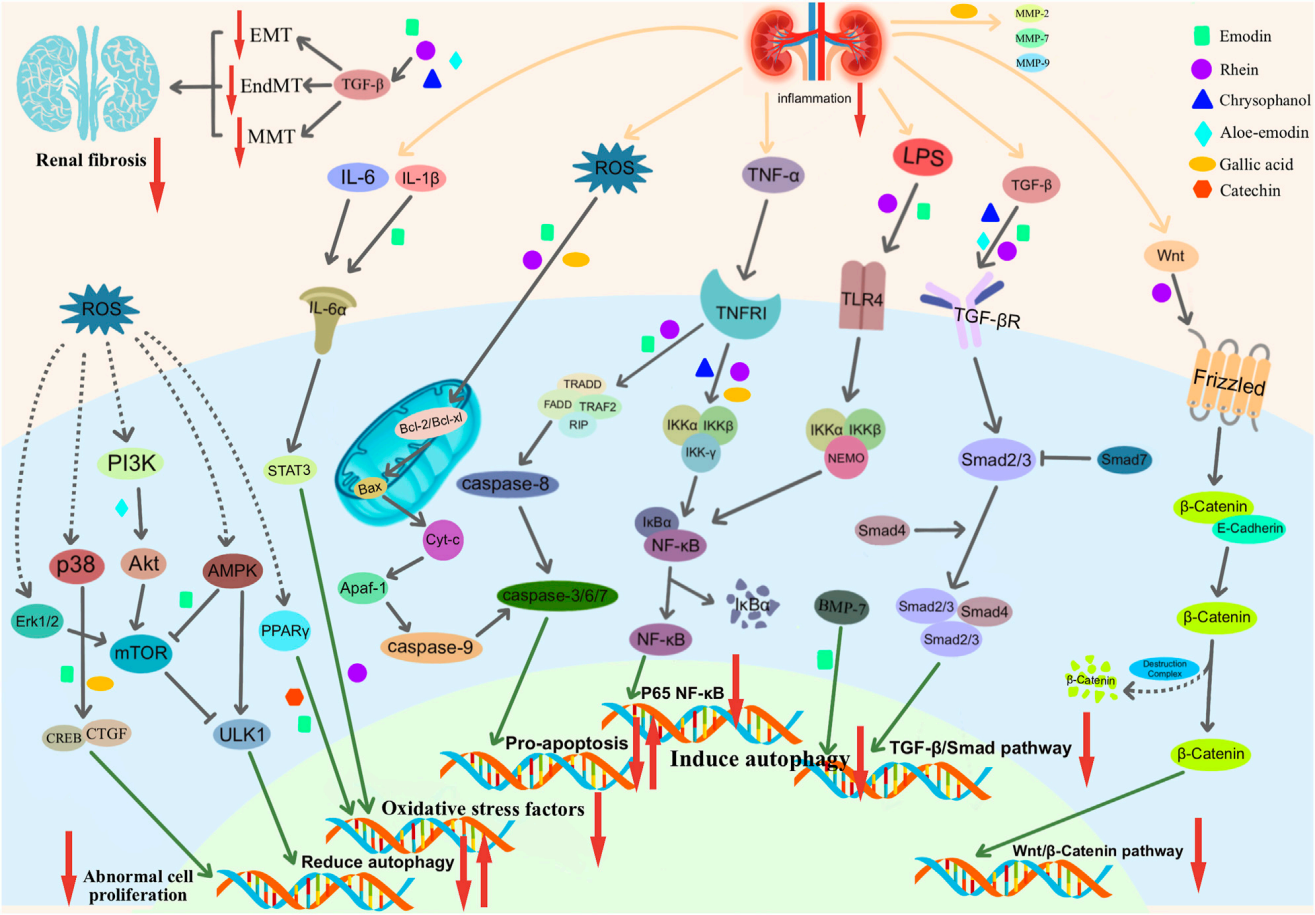
Pathways of action of natural extracts in rhubarb.

The natural components in *R. ribes L*. can inhibit renal fibrosis through multiple pathways. For example, the main targets of emodin are the TGF-β/Smad signaling pathway, p38/MAPK signaling pathway, MMP and AMPK/mTOR signaling pathway. Through the above pathways, emodin can ultimately reduce the levels of FN and ɑ-SMA, reduce the ECM deposition, and inhibit the expression of apoptosis-related factors. Rhein can inhibit renal fibrosis through the TGF-β/Smad signaling pathway, Wnt/β-catenin signaling pathway, DNA methylation, apoptosis and autophagy. Chrysophanol mainly acts on inflammation and the TGF-β/Smad signaling pathway and inhibits podocyte ECM, effectively alleviating fibrosis. Aloe-emodin showed a good protective effect on podocyte autophagy, which was mainly achieved by inhibiting the PI3K/Akt/mTOR signaling pathway. Other natural ingredients, such as gallic acid and catechins, primarily inhibit oxidative stress and collagen deposition. In general, the above natural ingredients for the treatment of renal fibrosis reflect the advantages of multiple targets, multiple pathways, extensive effects, strong pertinence, and considerable curative effects and have certain potential for exploration The molecular mechanism of action of natural ingredients against renal fibrosis can be summarized in (Figure 5; Table 1).

**TABLE 1:** Mechanism of natural components in rhubarb in the treatment of renal fibrosis.

Name	Cell/Animal model	Dosage	Pathway/Mechanism	References
Emodin	TECs	0–40 µM	LPS/TLR4/NF-κB signaling pathway	Zhu et al. (2012)
			IL-6mRNA, TLR4, TNFα	
	HG-induced mouse podocytes (*in vitro*)	30 μg/ml (*in vitro*)	desmin mRNA, nephrin mRNA, ILKmRNA	Chen et al. (2015)
	STZ-induced rat (*in vivo*)	20 mg/kg (*in vivo*)		
	TGF-β1-induced NRK-49F cells (*in vitro*)	0–50 μg/ml (*in vitro*)	TGF-β/Smad signaling pathway	Ma et al. (2018)
	5/6Nx-induced rat (*in vivo*)	0.3–1 mg/kg (*in vivo*)	TGF-β1mRNA, α-SMA, CTGF, Smurf2, Smad7, FN	
	TGF-β1-induced HK-2 cells (*in vitro*)	10–60 μM(*in vitro*)	TGF-β/Smad signaling pathway	Yang et al. (2020)
	UUO-induced rat (*in vivo*)	50 mg/kg (*in vivo*)	α-SMA, FN	
	HK-2 cells (*in vitro*)	100 μM(*in vitro*) 20–100 μM(*in vivo*)	BMP-7, Beclin1, LC3B,	Liu W. et al. (2021)
	5/6Nx-induced rat (*in vivo*)		α-SMA, FN, TGF-β	
	H/R-induced HK-2 cells (*in vitro*)	HK-2 cells	MAPK signaling pathway	Chen et al. (2017)
		10–80 µM	Bcl-2, Bax	
	Podocytes (*in vitro*)	20–40 μM(*in vitro*)	PERK/eIF2α signaling pathway	Tian et al. (2018)
	KK-Ay mouse, C57BL/6J mouse (*in vivo*)	40–80 mg/kg (*in vivo*)		
	MCs	50–100 mg/L	p38/MAPK signaling pathway	Wang et al. (2007)
			P38mRNA, P-P38, P-MKK3/6, P-MKK4	
	HBZY-1	10–60 μM	p38/MAPK signaling pathway	Li et al. (2009)
			CREB, CTGF	
	RMC cells	10–40 μM	Bax, caspase	Xu et al. (2016b)
	HBZY-1 (*in vitro*)	50 μM(*in vitro*)	cFLIP, caspase-8, caspase-3, caspase-9, PCNA	Gao et al. (2014)
	STZ-induced rat (*in vivo*)	30 mg/kg (*in vivo*)		
	MCs	50–100 mg/L	p38, PPARγmRNA	Liu et al. (2009)
Rhein	UUO-induced rat	150 mg/kg	AMPK/mTOR signaling pathway	Chen et al. (2019)
			Collagen I, STAT3, Bax, Bcl2, α-SMA	
	HK-2 cells (*in vitro*)	100–150 mg/kg	SIRT3/FOXO3a signaling pathway	Wu et al. (2020)
	5/6Nx-induced rat (*in vivo*)		ROS	
	CGN-induced rat	100 mg/kg	NF-κB signaling pathway	Chen et al. (2022)
			TNF-α, IL-1β, IL-6, ICAM-1, -IκBα	
	LPS-induced HK-2 (*in vitro*)	25–50 μg/ml	NF-κB signaling pathway	Liu M. et al. (2021)
	5/6Nx-induced rat (*in vivo*)		TNF, IL 6, MCP-1, IκBα	
	NRK-49F cells (*in vitro*)	0.01–1 ng/ml (*in vitro*)	α-SMA, FN, TGF-β1	He et al. (2011)
	UUO-induced mouse (*in vivo*)	150 mg/kg/d (*in vivo*)		
	db/db mouse	120 mg/kg	wnt/β-catenin signaling pathway	Duan et al. (2016)
			p-GSK-3b/tGSK-3b, wnt1	
	RAW, HEK293, HK-2, THP-1 cells (*in vitro*)	120 mg/kg	NF-κB signaling pathway	Bi et al. (2018)
	AKI-induced mouse (*in vivo*)		Klotho, TLR4	
	TGF-β-induced HK-2 cells (*in vitro*)	10 μg/ml	TGF-β/Smad signaling pathway, Wnt/β-catenin signaling pathway	Zhang et al. (2016)
	UUO-induced mouse (*in vivo*)		Klotho, DNMT	
	RMC cells	10–40 μM	Bax, caspase3	Xu et al. (2016a)
Chrysophanol	TGF-β1-induced HK-2 cells (*in vitro*)	0–100 μM	TGF-β/Smad signaling pathway, NF-κB signaling pathway	Dou et al. (2020)
	UUO-induced mouse (*in vivo*)	10–40 mg/kg (*in vivo*)	TβRI, Smurf2, FN, TGF-β1,ɑ-SMA	
	AB8/13 cells (*in vitro*)	40 μM(*in vitro*)	TGF-β/Smad signaling pathway	Guo et al. (2020)
	C57BL/6 mouse (*in vivo*)	50–100 mg/kg (*in vivo*)	TNF-ɑ、IL-6、IL-1β	
	HK-2 cells (*in vitro*)	5–20 μM(*in vitro*)	NF-κB signaling pathway	Ma et al. (2021)
	C57BL/6 mouse (*in vivo*)	20–40 mg/kg (*in vivo*)	Bax, Bcl2, CXCL2, TNF-α、IL-1β、IL-6, p53, p65	
Aloe-emodin	HK-2 cells (*in vitro*)	20–100 μM(*in vitro*)	PI3K/Akt/mTOR signaling pathway	Dou et al. (2019)
	C57BL/6 mouse (*in vivo*)	10 mg/kg (*in vivo*)	TGF-β1	
Gallic acid	Glyoxal-induced rat	100 mg/Kg	MMP-2, MMP-9, ROS	Yousuf and Vellaichamy (2015)
	HFD/STZ-induced rat	25–50 mg/kg	p38 MAPK signaling pathway, NF-κB signaling pathway	Ahad et al. (2015)
			IL-1β, IL-6, TNF-α	
Catechin	Wistar rats	50–100 mg/kg	ROS	Anjaneyulu et al. (2003)

Abbreviation; CKD, chronic kidney disease; TGF-β, Transforming Growth Factor-β; TGF-βR I, TGF-β1, receptor I; wnt, Wingless/Integrated; AngⅡ, Angiotensin II; NF-κB, Nuclear Factor kappa-B; IL-1, Interleukin-1; TNF-ɑ, Tumor Necrosis Factor-α; ECM, extracellular matrix; EMT, Epithelial-Mesenchymal Transition; ɑ-SMA, alpha-Smooth Muscle Actin; EndMT, Endothelial-Mesenchymal Transition; FSP-1, Fibroblast Specific Protein1; Smad, *drosophila* mothers against decapentaplegic protein; IL-1β, Interleukin-1β; PDGF, platelet derived growth factor; MIP-1a, Macrophage Inflammatory Protein 1α; TWEAK, TNF-like weak inducer of apoptosis; mi-RNA, Micro-RNA; MAPK, Mitogen-Activated Protein Kinase; PI3K, Phosphatidylin-ositol-3-Kinase; Akt, protein kinase B; mtor, Mammalian Target Of rapamycin; LEF, late expression factoc gene; MMP, matrix metalloproteinase; PAI-1, Plasminogen Activator Inhibitor-1; TIMP, tissue inhibitor of matrix metalloproteinases; CTGF, connective tissue growth factor; ROS, reactive oxygen species; Smac, second mitochondria-derived activator of caspases; Apaf-1, Apoptotic protease activating factor-1; TRADD, TNFR-associated death domain protein; FADD, Fas-associating protein with a novel death domain; IKK, inhibitor of kappa B kinase; Ub, Ubiquitin; NBR1, Neighbor Of BRCA1 Gene 1; mTOR, mammalian Target Of Rapamycin; MAPK, Mitogen-Activated Protein Kinase; Erk, Extracellular Regulated protein Kinases; AMPK, Adenosine 5‘-Monophosphate (AMP)-activated Protein Kinase; JNK, c-Jun N-terminal kinase; Bcl-2, B-cell lymphoma-2; TSC, Tuberous sclerosis subunit 1/2; CpG, cytosine guanine; AKI, acute kdney injury; CKD, chronic kidney disease; DN, diabetic nephropathy; DKD, diabetic kidney damage; DNMT1, DNA, methyltransferase 1; NOS3, Nitric Oxide Synthase 3; TGFB3, Transforming Growth Factor B3; RASAL1, Ras protein activator like-1; HATs, Histone Acetyltransferase; HDAC, histone deacetylase; UUO, unilateral ureteral obstruction; TSA, Trichostatin A; FK228, Romidepsin; MS-275, Entinostat; EGRF, epidermal growth factor receptor; STAT3, signal transducer and activator of transcription 3; H3K4me, Histone Lysine 4 Methylation; FN, fibronectin; TECs, Tubular Epithelial cells; Smurf2, Smad ubiquitin regulatory factor2; BMP-7, Bone Borphogenetic Protein 7; p62, P62-mediated mitophagy inducer; LC3B, LC3 protein B isoform; MCs, Mesangial Cells; P- MKK3/6, Phosphorylated mitogen-activated protein kinase MKK3/6 antibody; CREB, cAMP-response element binding protein; KKay, type 2 diabetic mice; HG, high glucose; PERK, protein kinase RNA–like endoplasmic reticulum kinase; Scr, Serum creatinine; BUN, blood urea nitrogen; Caspase, cysteinyl aspartate specific proteinase; FLICE, Caspase 8; STZ, streptozotocin; db/db, diabetic rat; SIRT3, Silent Information Regulator 3; PPAR-γ, Peroxisome Proliferator- Activated Receptor γ; HK-2, Human proximal tubular epithelial cell line.

Although studies have shown that the natural components of *R. ribes L*. have extensive protective effects on various targets of renal fibrosis, there are also certain limitations. For example, some natural ingredients have poor solubility, low bioavailability, poor intestinal absorption, strong dose dependence when intervening in some targets, hepatotoxicity and nephrotoxicity (Dong et al., 2016; Xie et al., 2019; Dong et al., 2020; Li et al., 2021). Most of the above conclusions are based on animal experiments, and there is a lack of their use in clinical practice. It is imminent to find efficient antifibrotic drugs. In the future, large-scale clinical studies can be carried out that focus on improving bioavailability and revealing the relationship between dose and nephrotoxicity.

Although modern pharmacological studies have proven that the natural components in *R. ribes L*. have certain toxicity to the liver and kidney, it is interesting that *R. ribes L*. does not show obvious toxicity in general. At a certain dose, it has a protective effect on the liver and kidney (Xiang et al., 2020; Zhuang et al., 2020). This may be related to factors such as the route of administration, individual differences, or interactions between several natural components. In addition, studies have found that the antifibrotic efficacy of some of the above natural products is significantly increased when combined. For example, curcumin and rhein showed synergistic effects when combined, and the therapeutic effect increased (He et al., 2020). The combined application of emodin and HGF increased the inhibitory effect on the TGF-β/Smad signaling pathway (Yang et al., 2020). This suggests that the combination of natural products with other antifibrotic drugs can increase efficacy and reduce toxicity. In terms of drug extraction, the subcritical water extraction method discovered in recent years has certain advantages in the extraction of natural products (Cheng et al., 2021). The application of this method to the extraction of anthraquinones may increase the extraction rate and utilization rate. In general, understanding the dose or combination of drugs may be the key to increasing the antifibrotic efficacy and reducing toxicity, and emerging extraction technology may have certain potential in improving bioavailability.

In conclusion, the extensive anti-renal fibrosis effect of the natural components in *R. ribes L*. provides information for the development of new drugs for renal fibrosis, and future in-depth research may find new targets and new ways to treat renal fibrosis.
